# Focus on bone healing: new strategies for improvement of bone healing

**DOI:** 10.1007/s00068-020-01344-x

**Published:** 2020-04-01

**Authors:** Dirk Henrich

**Affiliations:** Department of Trauma-, Hand- and Reconstructive Surgery, University Hospital Frankfurt, Theodor Stern-Kai 7, 60590 Frankfurt, Germany

Dear Reader,

It is a great pleasure for me to present the current special issue of “Focus on bone healing: new strategies for improvement of bone healing”. The treatment of large bone defects is still challenging despite advances in surgical techniques and improvements in clinical procedures, and the non-union rate has not been significantly reduced despite all efforts so far. Furthermore, the standard procedure of taking bone grafts from the iliac crest is a painful procedure and alternatives are warranted. It is therefore important that new treatment strategies are developed and evaluated through basic and applied research. The articles compiled here show in particular the broad range of current research and also provide new impulses for the development of new treatment concepts.

A very interesting approach that has received little attention so far is the use of weak electrical currents to stimulate bone healing. Although electrostimulation has supported bone healing in various experimental models, it is not widely established in clinical practice. In two complementary systematic reviews, Prof. John Barker’s research group summarizes the current state of knowledge on electrostimulation in the field of osteogenesis at the cellular and molecular level [1] and corresponding animal experimental and clinical analyses [2]. It is concluded that electrostimulation has a great potential but currently high costs and inconsistent results contrast with a wider dissemination of this approach.

Another promising method for stimulating bone healing is the use of autologous cells with regenerative potential. Mononuclear bone marrow cells (BMC) have proven to be easy to handle and in combination with bone replacement materials, safe and effective for bone defect healing. However, no systematic dose–response data are available for these cells in the field of bone defect healing. This gap is closed by Janko et al. [3], who identified an optimal dose range where relevant bone healing parameters are significantly improved without leading to an increase in foreign body reactions compared to the bone substitute material without cells. The results of this analysis could be of high relevance for the planning of corresponding clinical trials.

Bone graft substitutes play an important role in the therapy of large bone defects. They serve as carrier material e.g. for autologous bone marrow and/or regenerative cells. For a short time now, the 3D printing process has made it possible to develop new geometries while taking mechanical and biological aspects into account. Furthermore, these materials can be coated with potentially pro-osteogenic substances. Klein et al. [4] investigated the effect of a bone-sialoprotein coating on a 3D-printed mineral scaffold in a rat bone defect model. A tendency towards improved bone ingrowth was observed after 4 weeks, an effect that was no longer detectable after 8 weeks of healing. It is concluded that the possible role of bone-sialoprotein for bone regeneration remains unclear.

In addition to novel procedures such as electrostimulation, cell therapy and the development of bone replacement materials, the use of established osteoanabolic substances is another possibility to support bone healing. Leiblein et al. [5] compared known bone anabolic substances in typical therapeutic application concentrations, thereby using a rat model of distraction osteogenesis. They were able to demonstrate that most of the test substances led to a significant increase in bone formation and biomechanical load capacity compared to the control. It is concluded that these substances, as effective stimulants of bone formation, may be suitable to reduce the consolidation time in patients.

The induced membrane technique according to Masquelet is a two-step procedure that allows the healing of even large bone defects. In the first surgical step, a membrane pocket is induced around the bone defect using PMMA spacers, which is filled with a graft, e.g. cancellous bone, in the second surgical step. The time between the two operations, during which the membrane is formed as part of a foreign body reaction, is not clearly defined and can vary greatly in clinical practice. Whether the histological and biological properties of the induced membrane depend on the maturation time was investigated by Gindraux et al. [6] using corresponding membrane samples from patients. Interestingly, the content and osteogenic potential of membrane-bound mesenchymal stem cells as well as the histological membrane structure were preserved over time, so that a prolonged waiting time between operations does probably not affect the pro-osteogenic properties of the induced membrane.

A completely innovative link between basic research and clinical-operative research is offered in the work of Verboket et al. [7]. Here, the possibility of significantly shortening the two-stage induced membrane technique described by Masquelet using a human acellular dermis as a replacement for the induced membrane is demonstrated. In a femoral defect model of the rat, the newly developed one-step technique was compared with the two-step induced membrane technique with respect to different bone healing parameters. Using the single-stage technique, a bone healing result equivalent to the two-stage procedure was achieved. Both groups also showed a significantly improved bone healing compared to the control group, which was treated with cancellous bone alone. After further evaluation, this innovative procedure may find its way into everyday clinical practice and significantly reduce the duration of treatment of large bone defects.

I hope you enjoy reading the articles on “Focus on bone healing: new strategies for improvement of bone healing” and that they will inspire you to develop and evaluate new treatment concepts.

With kind regards

Dirk Henrich.
